# Effect of the Pulsed Electromagnetic Field (PEMF) on Dental Implants Stability: A Randomized Controlled Clinical Trial

**DOI:** 10.3390/ma13071667

**Published:** 2020-04-03

**Authors:** Bhukya P. Nayak, Oleg Dolkart, Parth Satwalekar, Yeramala P. Kumar, Anam Chandrasekar, Ophir Fromovich, Elad Yakobson, Shlomo Barak, Ulisses Dayube, Jamil A. Shibli

**Affiliations:** 1 SVS Institute of Dental Sciences, Mahabubnagar, Kaloji Narayana Rao University of Health Sciences, Telangana 509001, India; bhukyaparashuramnayak@gmail.com (B.P.N.); parthsat@hotmail.com (P.S.); yeramala.praveen@gmail.com (Y.P.K.); anamchandrasekar@gmail.com (A.C.); 2 Division of Orthopedic Surgery, Tel Aviv Sourasky Medical Center, Tel Aviv University Sackler Faculty of Medicine, Tel Aviv 6423906, Israel; 3 Privet Practice, Nordau 83, Tel Aviv 62381, Israel; info@identalmaster.com; 4 Magdent Ltd., Bnei-Brak 5120109, Israel; elad@magdentmed.com (E.Y.); barak@magdentmed.com (S.B.); 5 Department of Periodontology and Oral Implantology, Dental Research Division, University of Guarulhos, 07023-040 Guarulhos, SP, Brazil; dayubecd@globo.com (U.D.); jshibli@ung.br (J.A.S.)

**Keywords:** pulsed electromagnetic field, osseointegration, implant stability, healing abutment

## Abstract

A pulsed electromagnetic field (PEMF) has been shown to contribute to heightening bone regeneration in a range of clinical areas, including dentistry. Due to the scarcity of studies using PEMF in oral implantology, the present experiment scrutinized the effect of PEMF can lead to improving the stability of the implant. A total of 19 subjects (40 implants in total) were selected to participate in the current study and were randomly allocated to either the PEMF group or control group. Subjects in the PEMF group received an activated miniaturized electromagnetic device (MED) while the control group received a sham healing cup. Implants stability was assessed by resonance frequency analyses (RFA) via implant stability quotient (ISQ) calculations. RFA were recorded as following: immediately after procedure, and then 2, 4, 6, 8 and 12 weeks later. Radiographic analysis was performed at baseline, 6 and 12 weeks after implant placement. Proinflammatory cytokines were evaluated in peri-implant crevicular fluid (PICF). The PEMF group presented higher ISQ mean values when compared to the control group. The primary stability time frame (the first 2 weeks) MED group depicted an increase in stability of 6.8%, compared to a decrease of 7.6% in the control group related to the baseline. An overall stability increase of 13% was found in MED treated group (*p* = 0.02), in contrast, the overall stability in the control group decreased by 2% (*p* = 0.008). TNF-α concentration during first 4 weeks was lower in the MED treated group. The data strongly suggests that MED generated continuing a PEMF may be considered as a new way to stimulate the stability of the implants at the early healing period.

## 1. Introduction

Dental implants have become a popular solution as a prosthetic treatment and have high success rates [1]. However, the time for functional loading once the implant is osseointegrated still remains long for the vast majority of implants. However, some recently introduced implants can be loaded much earlier than in classical approaches [2]. This is important since the waiting period is considered to be uncomfortable by the vast majority of patients and can adversely influence their quality of life [3,4].

One of the key factors affecting the final osseointegration is primary implant stability. There is evidence that both tissue quantity and quality at the interface are crucial to implant primary stability and thus the success and comfort of immediately loaded implants [5]. Primary stability involves the way the implant engages mechanically with the surrounding bone, whereas secondary (biological) stability is determined by bone regeneration and remodeling processes [6,7]. Secondary stability has been positively associated with a successful primary stability [6]. Thus, there is clearly an unmet need for additional treatment modalities to overcome problems of poor bone quality by fostering osteogenesis, which in turn can reduce the loading time.

PEMF or pulsed electromagnetic field has been shown to contribute to heightening the regeneration of bone tissue in a range of clinical areas. PEMFs affect cell differentiation and proliferation by influencing several metabolic pathways, reliant on the lineage and maturation stage [8]. In addition, PEMFs contribute to bone tissue formation followed by a demineralized bone matrix and stimulate fracture healing, possibly through the action of progenitors that are already committed towards bone [8].

In dentistry, PEMF stimulation may constitute a valuable new technique to encourage bone formation, ingrowth of bone on dental implants, and increased bone stock. This may help decrease the time to osseointegration and allow patients to return to normal loaded eating activities sooner [9]. A recently published study [10] reported that in rabbits, PEMF devices stimulated early osseointegration and ingrowth of bone onto dental implants by more than 3 fold.

The miniaturized electromagnetic device (MED; Magdent ltd, Bnei-Brak, Israel) is shaped like a dental healing abutment. It houses microelectronic modules that generate PEMF to improve bone formation after a dental implant procedure. The MED is screwed onto the exposed portion of the dental implant using standard routine protocols, in place of the standard healing abutment. A recent prospective case study showed that MED-abutment implants outperformed standard implants in the initial phase of healing [11].

This randomized clinical study evaluated the effects of PEMF on implant stability through resonance frequency analyses (RFA). Based on recently published in-vitro [8] and in-vivo [10] studies results, it was hypothesized that by enhancing the activity and proliferation of osteoblasts, the MED treated group, as compared to controls, would show superior secondary stability starting earlier in the implant integration process, thus resulting in better overall stability. The influence on some proinflammatory cytokines was also evaluated.

## 2. Materials and Methods 

The Institutional Ethical Committee (SVS Institute of Dental Sciences, SVSIDS/PROSTH/3/2016) approved the study protocol. All patients gave their informed consent before the commencement of the study. A total of 19 partially edentulous the subjects (10 females and 9 males, average age 37 ± 9.7 years) were included in this study. The alveolar ridge must present at least 6 mm wide and 10 mm length. Forty implants (Touareg™-S, conical connection, Al_2_O_3_ blasted/acid etched surface treatment, ADIN Dental Implant Systems Ltd., Afula, Israel) measuring 3.75 mm in diameter and 10–11.5 mm long were inserted in the maxilla and mandible of 19 patients. Patients were excluded if the following were present: a lack of bone tissue volume, bruxism, smoking, high alcohol intake current or previous head and neck radiation therapy, chemotherapy, a diagnosis of an uncontrolled systemic disease or immunodeficiency and inflammatory or autoimmune diseases.

### 2.1. Surgical Procedures 

All surgical procedures were performed in outpatient clinics under local anesthesia. Delayed implant placement protocol was followed. Implants were placed at 3–6 months after the tooth extraction. Implants were placed according to standard protocols. To standardize the bone-level at surgery as much as possible, each implant was placed at the bone level. Prophylactic antibiotics were prescribed as customary in our institution. All patients received amoxicillin 1 g preoperatively, and then 500 mg TID for five days. After implantation, two MED devices (half active and half non-active sham MED devices) were randomly inserted into the dental implants. An independent investigator that did not participate of inclusion neither treatment of the subjects, assigned to one of the two treatment groups by means of a computer-generated random sequence (Random Allocation Software).

**Figure 1:**
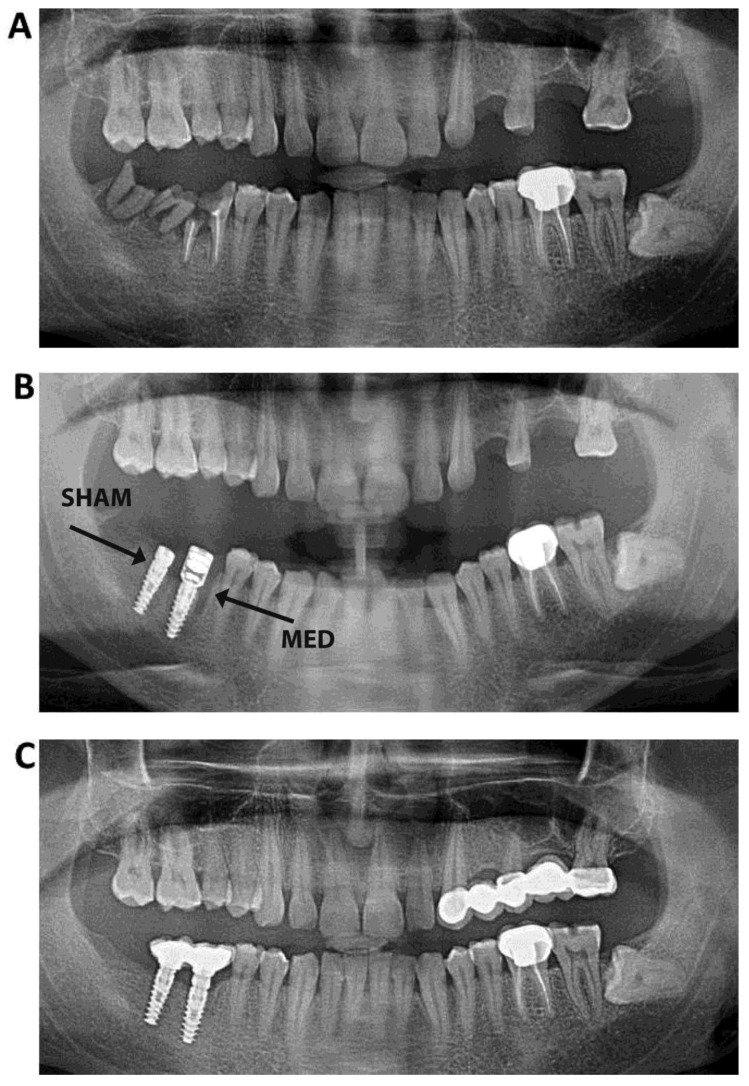
Radiographic appearance of a miniaturized electromagnetic device (MED; test) and sham (control) healing abutments, (**A**) before the implantation; (**B**) 6 weeks post-implantation and (**C**) 12 weeks post-implantation.

The implants were placed in the same quadrant or bilaterally on the contralateral teeth. PEMF generated by MED is active within the radius of 2 mm, therefore there was no effects on the adjacent tooth. The distance radiance of the distal adjacent tooth or adjacent implant was at least 3 mm. PEMF treatment was administered via the MED at an exposure ratio of 1/500–1/5000, intensity: 0.05–0.5 mT and frequency: 10–50 kHz for 30 days (Figure 1).

### 2.2. Post-Operative Procedures

All the patients received detailed training in post-operative dental hygiene and care. They followed standard instructions issued by the clinic for implanted patients, including rinsing twice a day with 0.1–0.2% chlorhexidine digluconate.

On each follow-up visit each participant was evaluated for problems associated with the implants. In particular this included the degree of healing and the presence of local inflammation of the soft tissue around the implant.

### 2.3. Radiographic Evaluation

Digital images of dental implants were obtained using the parallelism technique at baseline, 6 and 12 weeks for all implants. The measurements of the distance between the abutment and the peri-implant bone at the distal and mesial sites were made using Image Tool 3.0. Software [12]. These measurements, performed in duplicate, were taken by a single previously trained examiner (U.D.).

### 2.4. Resonance Frequency Analysis (RFA)

RFA was evaluated with an Osstell device (Osstell Mentor^®^, Integration diagnostics AB, Sävedalen, Sweden) after dental implant placement (Week 0) and then at weeks 2, 4, 6, 8 and 12 postoperatively. RFA measurements were made in two perpendicular directions (mesio-distal (M–D) and buco-lingual (B–L)), twice in each direction. The RFA yielded the implant stability quotient (ISQ), namely the change in ISQ from the mean baseline measurement for each implant. One investigator (P.N.) conducted all the measurements.

### 2.5. Inflammatory Mediator Levels 

After removing the healing abutments, the implant was isolated with cotton rolls and the area was dried. A standardized volume of 3–5 µL peri-implant crevicular fluid (PICF) from each implant site was collected using calibrated pipettes positioned extra-crevicularly on the margin of the gingiva. PICF was moved to an eppendorf tube. The samples were centrifuged at 3000 *g* for 5 min, and the supernatants were stored at −80 °C. Levels of IL-1β and TNF-α were measured in PICF. Samples were collected postoperatively at the following time points: 2 weeks, 4 weeks, 3 months, 6 months and 12 months.

### 2.6. Analysis of IL1-β and TNF-α

The levels of pro-inflammatory cytokines interleukin (IL)-1 were measured by ELISA using human IL-1 ELISA kit (Invitrogen, cat. no. KHC0011; Thermo Fisher Scientific, Inc., Waltham, MA, USA), and human TNF-α ELISA kit (Invitrogen, cat. no. KHC3011; Thermo Fisher Scientific, Inc., Waltham, MA, USA) according to the manufacturer’s instructions. For the antibody-coated wells, 96-well plates were used. Sixteen wells for the standard curve and 80 for study samples were used. Each standard curve and samples were tested in duplicates. The microtiter plate that was furnished with this kit was precoated with specific antibodies (either to IL-1β and TNF-α). Standards or samples from PICF fluid were added to the appropriate microtiter plate wells with a biotin-conjugated polyclonal antibody. Avidin conjugated to horseradish peroxidase was added to each microplate well. These were incubated according to the manufacturer’s instructions. A tetramethylbenzidine substrate solution was then added to each well. Only wells containing lung-originated IL-1β and TNF-α biotin-conjugated antibody and enzyme-conjugated avidin exhibited a change in substance color. The enzyme–substrate reaction was ended by adding a sulfuric acid solution, which halted color transformation, thus enabling spectrophotometric color determination at a wavelength of 450 ± 2 nm. The IL-1β and TNF-α levels in the samples were then established by comparison of the optical density (OD) of the samples with the standard curve OD.

### 2.7. Statistical Analyses

Power analysis study indicated that a sample size of 20 implants in each group would yield 80% power, at a 95% confidence interval, with *p* < 0.05 using specific software (SPSS SamplePower package, IBM, Armonk, NY, USA).

The radiographic measurements were subjected to either a Mann-Whitney (intergroup comparison) or a Wilcoxon test (intragroup comparison). SPSS version 21 was implemented to calculate the descriptive statistics; a repeated measures ANOVA and paired *t*-test at α = 0.05 were run where appropriate.

Changes in cytokine levels in both groups were analyzed by using an analysis of variance (ANOVA), and the Tukey post hoc test were used. If homogeneity of variance was significant, the Kruskal-Wallis test was used; results are presented as mean ± SD of the mean. 

## 3. Results

Overall, 40 implants were placed in the jaws of 19 subjects. Eighteen patients had two implants (one with an active MED and one sham), one patient received four implants (two MEDs and two sham devices). As for the MED treated group: 3 implants were inserted in the D1 bone type, 10 implants in D2, 5 implants in D3 and 2 implants in the D4 bone type. In control group, the implant distribution was as follows: 3 implants were inserted in the D1 bone type, 11 implants in D2, 6 implants in D3 and no implants were inserted in the D4 bone type. All patients presented a sufficient bone volume for implant placement with no need of bone augmentation. After implantation, the patients reported no or only minor discomfort at the surgical sites.

### 3.1. Implant Stability

**Figure 2:**
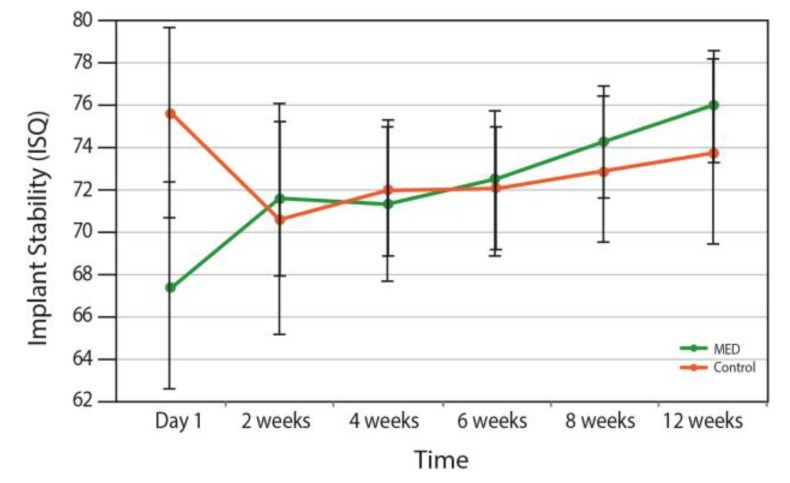
Changes in implant stability in the MED and control groups over time.

Figure 2 shows the changes over time on dental implants stability in the groups. The intergroup analyses failed to detect any difference over time between groups (*p* = 0.577). 

**Figure 3:**
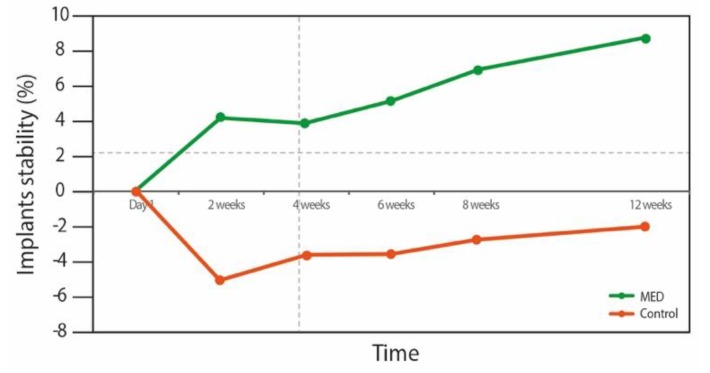
Intergroup repeated measures ANOVA analyses. Pairwise comparison of mean implant stability at different time points for the MED and control groups.

However, intragroup analyses found a significant improvement within the MED treated group (Figure 3). During the first two weeks (primary stability time frame) the MED treated group presented an increase in the stability of 6.8%, whereas stability in the control group decreased by 7.6% compared to the baseline. As of week, 2, however, both groups demonstrated an increase in stability values. This stability increase was only statistically significant at all-time points in the MED treated group (*p* < 0.05). Implants from the test group presented an increase of 13% (*p* = 0.02), when control group implants depicted an overall decrease in stability of 2% (*p* = 0.008).

### 3.2. Radiographic Evaluation

**Figure 4:**
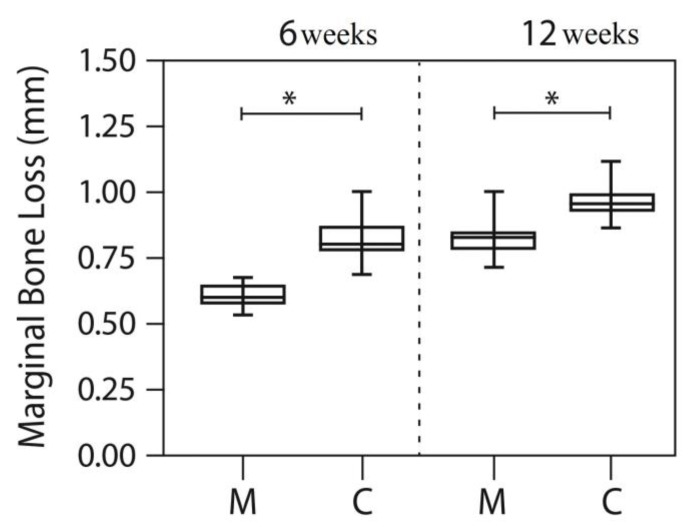
Box-plot with min–max values for the control (C) and test (MED) groups at 6 and 12 weeks using the Mann-Whitney test, *p < 0.05.

After averaging the mesial and distal measurements of all dental implants, the means for peri-implant bone loss at 6 and 12 weeks were lower in the MED group than in the control group (without PEMF; Mann-Whitney *p* < 0.05). Figure 4 and Table 1 depicts the means for the MED and sham groups at both time periods. At 12 weeks, the peri-implant bone loss was higher than at 6 weeks for both groups (Wilcoxon test, *p* < 0.05).

### 3.3. Proinflammatory Mediators

**Figure 5:**
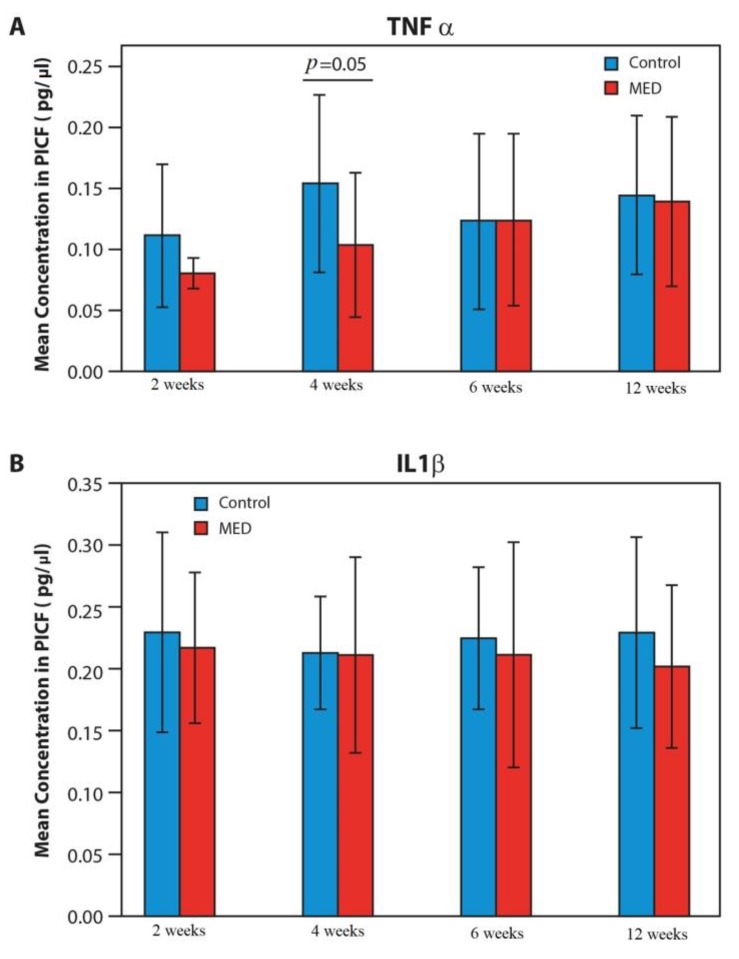
Changes in cytokine levels in the MED and control groups over time, an analysis of variance (ANOVA), and the Tukey post hoc test that followed ANOVA, were used. If homogeneity of variance was significant, the Kruskal–Wallis test was used; results are presented as mean ± SD of the mean. *p < 0.05 control vs. MED.

There was a rapid increase of 30% in the mean TNF-α concentration during the first 4 weeks in the control group as compared to a slight increase (15%) in the MED treated group. From week 4 to week 12, the TNF-α concentration remained similar in the two groups (Figure 5).

During the first two weeks, levels of IL-1β were high. The MED treated group exhibited a slight decrease in IL-1β levels between weeks 2 and 4 (20%). Levels in the control group declined gradually from week 2 to 8, followed by an increase as of week 12 when the peak concentrations of IL-1β were found. No such peak was seen in the MED treated group (Figure 5).

## 4. Discussion

The principal result emerging from the intragroup analyses in this randomized clinical study, revealed that MED treated implants presented an increase of 13% (*p* = 0.02) of the stability, in contrast to the control group, which exhibited an overall decrease in stability of 2% (*p* = 0.008). During the first two weeks the test group exhibited an increase in stability of 6.8%, whereas stability in the control group decreased by 7.6% as compared to the baseline. Radiographic evaluation indicated a significantly lower marginal bone loss in the test group at 6 and 12 weeks.

Primary stability is of utmost importance as it determines the overall stability and survival of the implants in the long term [13]. Osseointegration is a complex ongoing process, which involves the replacement of primary stability by secondary stability. Total stability is the sum of the primary stability that decreases over time, and secondary stability is that which increases. A temporary drop in total stability, which is typically found in the first 3–4 weeks after implant installation, is a well-known clinical phenomenon and is related to the decline in primary stability [14]. Thus good total stability with a less marked stability dip, requires a good balance between cortical and trabecular bone around the newly placed implant [14].

In this study, in the group of implanted patients treated with the MED generated PEMF, no stability drop was noted. This resulted in superior total stability as measured by RFA at the 12 weeks follow up, compared to the control group. During the first week, control group showed higher stability (not statistically significant), where it might be assumed that this may be related to the slightly higher primary stability depending on the way the implant engages mechanically with the surrounding bone.

Recently published in-vitro [8] and in-vivo [10] data along with these results, thus strongly suggest that by enhancing the activity and proliferation of osteoblasts, MED generated PEMF was able to shift the balance between bone resorption and formation in favor of the formation process. This resulted in superior secondary stability that began earlier in the implant integration process in the MED treated group.

A better grasp of the mechanism of implant integration, and specifically the inflammatory response, is key to developing new treatment modalities to enhance osteointegration and the subsequent stability of the implants. Clearly, there are crucial lessons to be drawn for dentistry. Ferroni et al. [8] found that PEMF enhanced cell proliferation, adhesion and the osteogenic commitment of MSCs, even in inflammatory conditions. Their evidence indicated that PEMFs heightened anti-inflammatory cytokine, such as IL-10 expression, and lessened the expression of the pro-inflammatory cytokine IL-1. In the current clinical study, cytokines levels (TNF-α and IL1-β) were lower in the MED treated group.

A study by Bielemann et al. [15] examined the relationships between clinical features, implant stability and cytokine levels in PICF during early bone healing after implant surgery. Their conclusion was that the first 2 weeks are crucial for a good plaque and gingival index. The probing depth stabilized on the 8th week, whereas the ISQ values decreased a month into healing. They argued that the variability in cytokine concentrations and their major role could be attributed to the nature of the balance at different time periods in the healing process. These findings are consistent with the results here, implying that PEMF treatment positively influences implant stability by modulating the cytokine levels. A limitation of this study was the absence of microbiological data that could provide a better understanding of the immunological and clinical data. It could be speculated that PEMF modules the biofilm co-aggregation, influencing the cytokine levels as well as allowing a better microenvironment around dental implants [16].

## 5. Conclusions

This study showed the positive impact of PEMF around dental implants placed human jaws, suggesting that the use of MED devices (healing abutments) could be a useful procedure for improvement of clinical stability, at least in the initial stages of healing.

The data strongly suggests that MED generated continues PEMF may be considered as a new way to stimulate stability of the implants at the early healing period. These results should thus be taken into account when designing and implementing immediate and early loading protocols. Further studies with larger cohorts and a longer follow up period are recommended.

## Figures and Tables

**Table 1:** Results of the Bonferroni test for pairwise comparison of the mean implant stability at different time points in the MED and control groups (MD).

Time Points	Group	Mean Differences	Standard Error	* p Value	Change %
Day 1 vs. 2 weeks	MED	−4.1	2.6	0.12	+6.1
	Control	5	2.4	0.05	−6.7
Day 1 vs. 4 weeks	MED	−3.8	3	0.21	+6.2
	Control	3.5	1	0.003	−6.6
Day 1 vs. 6 weeks	MED	−5.1	3.1	0.12	+7.6
	Control	3.4	0.8	0.001	−4.6
Day 1 vs. 8 weeks	MED	−6.9	3.2	0.04	+10.2
	Control	2.7	0.6	0.00	−3.6
Day 1 vs. 12 weeks	MED	−8.5	3.3	0.02	+12
	Control	1.8	0.5	0.003	−2.5

## References

[1] Compton S.M., Clark D., Chan S., Kuc I., Wubie B.A., Levin L. (2017). Dental Implants in the Elderly Population: A Long-Term Follow-up. Int. J. Oral Maxillofac. Implant..

[2] Kuchler U., Chappuis V., Bornstein M.M., Siewczyk M., Gruber R., Maestre L., Buser D. (2017). Development of Implant Stability Quotient values of implants placed with simultaneous sinus floor elevation—Results of a prospective study with 109 implants. Clin. Oral Implant. Res..

[3] Omura Y., Kanazawa M., Sato D., Kasugai S., Minakuchi S. (2016). Comparison of patient-reported outcomes between immediately and conventionally loaded mandibular two-implant overdentures: A preliminary study. J. Prosthodont. Res..

[4] Penarrocha-Oltra D., Penarrocha-Diago M., Aloy-Prosper A., Covani U., Penarrocha M. (2015). Immediate Versus Conventional Loading of Complete-Arch Implant-Supported Prostheses in Mandibles with Failing Dentition: A Patient-Centered Controlled Prospective Study. Int. J. Prosthodont..

[5] Degidi M., Iezzi G., Perrotti V., Piattelli A. (2009). Comparative analysis of immediate functional loading and immediate nonfunctional loading to traditional healing periods: A 5-year follow-up of 550 dental implants. Clin. Implant. Dent. Relat. Res..

[6] Javed F., Ahmed H.B., Crespi R., Romanos G.E. (2013). Role of primary stability for successful osseointegration of dental implants: Factors of influence and evaluation. Interv. Med. Appl. Sci..

[7] Natali A.N., Carniel E.L., Pavan P.G. (2009). Investigation of viscoelastoplastic response of bone tissue in oral implants press fit process. J. Biomed. Mater. Res. B Appl. Biomater..

[8] Ferroni L., Gardin C., Dolkart O., Salai M., Barak S., Piattelli A., Amir-Barak H., Zavan B. (2018). Pulsed electromagnetic fields increase osteogenetic commitment of MSCs via the mTOR pathway in TNF-alpha mediated inflammatory conditions: An in-vitro study. Sci. Rep..

[9] Matsumoto H., Ochi M., Abiko Y., Hirose Y., Kaku T., Sakaguchi K. (2000). Pulsed electromagnetic fields promote bone formation around dental implants inserted into the femur of rabbits. Clin. Oral Implant. Res..

[10] Barak S., Neuman M., Iezzi G., Piattelli A., Perrotti V., Gabet Y. (2016). A new device for improving dental implants anchorage: A histological and micro-computed tomography study in the rabbit. Clin. Oral Implant. Res..

[11] Barak S., Matalon S., Dolkart O., Zavan B., Mortellaro C., Piattelli A. (2019). Miniaturized Electromagnetic Device Abutment Improves Stability of the Dental Implants. J. Craniofacial Surg..

[12] Shibli J.A., Melo L., Ferrari D.S., Figueiredo L.C., Faveri M., Feres M. (2018). Composition of supra- and subgingival biofilm of subjects with healthy and diseased implants. Clin. Oral Implant. Res..

[13] Albrektsson T., Zarb G.A. (1993). Current interpretations of the osseointegrated response: Clinical significance. Int. J. Prosthodont..

[14] Bosshardt D.D., Chappuis V., Buser D. (2000). Osseointegration of titanium, titanium alloy and zirconia dental implants: Current knowledge and open questions. Periodontology.

[15] Bielemann A.M., Marcello-Machado R.M., Leite F.R.M., Martinho F.C., Chagas-Junior O.L., Antoninha Del Bel Cury A., Faot F. (2018). Comparison between inflammation-related markers in peri-implant crevicular fluid and clinical parameters during osseointegration in edentulous jaws. Clin. Oral Investig..

[16] Barak S.D., Dolkart M., Faveri M., Bueno-Silva B., Soares G.M., Feres M., Shibli J.A. (2018). Antimicrobial effects of pulsed electromagnetic field: In vitro polymicrobial periodontal subgingival model. J. Clin. Periodontol..

